# Lumbopelvic Kinematics During Functional Tasks in a Chronic Low Back Pain Observational Cohort

**DOI:** 10.1002/jsp2.70117

**Published:** 2025-10-01

**Authors:** Kevin M. Bell, Rachel E. Roos, Zakiy Alfikri, William Anderst, Anna Bailes, William W. Clark, Harold A. Cook, Jessa Darwin, Marit Johnson, Gina P. McKernan, Sebastian Murati, Bambang Parmanto, Nam V. Vo, Leming Zhou, Gwendolyn A. Sowa

**Affiliations:** ^1^ Department of Bioengineering University of Pittsburgh Swanson School of Engineering Pittsburgh Pennsylvania USA; ^2^ Clinical and Translational Science Institute, University of Pittsburgh Pittsburgh Pennsylvania USA; ^3^ Department of Orthopaedic Surgery University of Pittsburgh School of Medicine Pittsburgh Pennsylvania USA; ^4^ Department of Physical Therapy University of Pittsburgh School of Health and Rehabilitation Sciences Pittsburgh Pennsylvania USA; ^5^ Department of Mechanical Engineering and Materials Science University of Pittsburgh Swanson School of Engineering Pittsburgh Pennsylvania USA; ^6^ Department of Physical Medicine and Rehabilitation University of Pittsburgh School of Medicine Pittsburgh Pennsylvania USA; ^7^ Department of Biomedical Informatics University of Pittsburgh School of Medicine Pittsburgh Pennsylvania USA; ^8^ Department of Health Information Management University of Pittsburgh School of Health and Rehabilitation Sciences Pittsburgh Pennsylvania USA; ^9^ Ferguson Laboratory for Orthopaedic and Spine Research, Bethel Musculoskeletal Research Center, Department of Orthopaedic Surgery University of Pittsburgh School of Medicine Pittsburgh Pennsylvania USA; ^10^ Department of Pathology University of Pittsburgh School of Medicine Pittsburgh Pennsylvania USA; ^11^ McGowan Institute University of Pittsburgh Pittsburgh Pennsylvania USA; ^12^ Intelligent Systems Program University of Pittsburgh School of Computing and Information Pittsburgh Pennsylvania USA

**Keywords:** acceleration, chronic low back pain, inertial measurement unit, kinematics, lumbopelvic rhythm, range of motion, velocity, wearable motion sensors

## Abstract

**Background:**

The University of Pittsburgh Mechanistic Research Center, entitled, “Low Back Pain: Biological, Biomechanical, Behavioral Phenotypes (LB^3^P),” is part of the National Institutes of Health's Helping to End Addiction Long‐term Initiative. LB^3^P conducted a prospective, observational cohort study to identify phenotypes from over 1000 participants with chronic low back pain (cLBP). This article reports findings from multi‐level inertial measurement unit (IMU) kinematic data collected during performance‐based tests obtained at the in‐person LB^3^P enrollment visit.

**Methods:**

Participants with cLBP were recruited and performed self‐paced and fast‐paced movements while wearing inertial measurement units (IMUs) placed over T1/T2, T12/L1, L5/S1, and along the right femur. For self‐paced tests: axial rotation (AR), lateral bending (LB), and flexion and extension (F/E), participants performed to their maximum range of motion (ROM), and for fast‐paced tests: combined rotation/flexion (CRF), AR, LB, flexion, five times sit to stand (5STS), and postural lifting strategy (PLS), participants performed at their maximum speed. ROM, velocity, acceleration, and lumbopelvic rhythm (LPR) were calculated for tests using IMU data. LPR was calculated as the ratio of absolute lumbar to hip movement and was extracted for each motion quartile (0%–25%, 25%–50%, 50%–75%, and 75%–100%) during neutral‐to‐flexion and neutral‐to‐extension.

**Results:**

Analysis of sensor data of 954 participants (58.6 ± 16.4 years old; 40% male and 60% female) revealed variable kinematic patterns across spinal and hip regions during isolated and functional movements. Noticeable variations were observed based on movement type, with the trunk region demonstrating predominant mobility during self‐paced movements like AR and LB, while the hip region played a critical role in functional tasks (CRF, 5STS, PLS). LPR evaluation indicated that individuals with cLBP typically adopt a hip‐dominant movement pattern, with slightly greater lumbar contributions during the initial phase of flexion. Sex and age analyses unveiled females generally exhibit greater ROM and higher velocities compared to males. Younger participants (< 60 years old) show more dynamic movement patterns, except in the hip region during F/E, where older (≥ 60) participants exhibited greater excursion.

**Conclusions:**

This study characterized spinal and hip movement in individuals with cLBP, focusing on ROM, velocity, acceleration, and LPR across a variety of self‐paced and functional tasks. The values established from this cohort provide a foundation for future cLBP phenotyping, offering insights to guide individualized treatment plans and inform clinical guidelines. These findings highlight the complex relationship between regional contributions, demographic factors, and movement demands in spinal and hip kinematics, emphasizing the need for person‐specific approaches to understanding the biomechanics of individuals with cLBP. Future research will expand this analysis by collecting the same metrics in asymptomatic individuals, enabling a more robust comparison to differentiate movement patterns and further refine the understanding of cLBP biomechanics. Future analyses will integrate these comprehensive kinematic data with the other study domains (behavioral and biological) to identify distinct cLBP phenotypes, which may serve as a basis for predicting treatment response and guiding personalized interventions.

## Graphical abstract

The University of Pittsburgh Mechanistic Research Center, entitled, “Low Back Pain: Biological, Biomechanical, Behavioral Phenotypes (LB3P),” is part of the National Institutes of Health's Helping to End Addiction Long‐term Initiative. LB3P conducted a prospective, observational cohort study to identify phenotypes of over 1000 participants with chronic low back pain (cLBP). This article reports on multi‐level inertial measurement unit (IMU) kinematic data collected from performance‐based tests obtained at the in‐person LB3P enrollment visit.

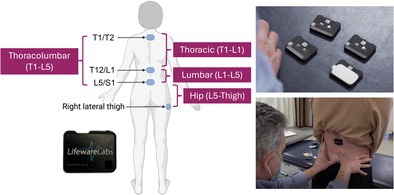

## 1 Introduction

Chronic low back pain (cLBP) has proven to be one of the most costly and difficult to treat chronic conditions of our time [1, 2, 3, 4]. cLBP is shaped by a complex combination of biopsychosocial factors, which have the potential to modify how an individual tolerates, generates, balances, and responds to tissue loading, which in turn impacts how an individual executes a movement [5]. Knowledge of these interrelationships can assist in classifying distinct patient subgroups (phenotypes) based on their movement patterns and other biopsychosocial characteristics. One important clinical characteristic that can be used for classification is the severity of physical impairment and the resulting activity limitations, which spine and hip kinematics can be used to predict [5]. Understanding how kinematic patterns relate to unique patient subgroups may aid in proposing targeted interventions and predicting clinical response.

Measuring functional spine and hip kinematics is often challenging in typical clinical settings due to limitations (practicality, accuracy, reliability) of standard clinical measurement tools such as goniometers, tape measures, and inclinometers [6, 7]. Optoelectronic motion capture is the current gold standard for quantifying spine kinematics [8]. Unfortunately, optical motion tracking requires dedicated space, expertise, and is expensive, making it impractical in many clinical settings. While many clinicians acknowledge the value of kinematic performance assessments, challenges in assessing them reliably in clinical environments often lead to reliance on skilled observation [9]. Recent advancements in the accessibility and reliability of wearable motion tracking technology (i.e., inertial measurement units, IMUs) have made them increasingly popular and are now considered necessary tools for many precision medicine applications [10]. Using wearable motion sensors to assess spine and hip kinematics during functional tasks enables objective assessment of patients' physical function and can provide insights into the mechanism of physical impairment and opportunities for tailored treatment regimens. Moreover, performance differences assessed with these sensors have been shown to reliably discriminate between populations with and without LBP [11, 12, 13, 14]. Individuals with cLBP exhibit distinct performance differences during dynamic movement tasks compared to asymptomatic controls. These differences include reduced lumbar ROM [15, 16, 17, 18, 19, 20, 21] and reduced velocity/acceleration [14, 15, 17, 20, 21, 22, 23, 24, 25, 26, 27]. Additionally, altered temporal and sequential patterns can be present during complex functional movements, such as walking or lifting. These findings underscore the potential of wearable motion sensors not only to enhance our understanding of kinematic differences between individuals with and without cLBP but also to provide novel insights into variability within the cLBP population.

While traditional assessments of spinal movement focus on a limited set of measures, such as range of motion (ROM) and postural assessment, this study takes a more comprehensive approach by using wearable motion sensors to examine a broad range of kinematic metrics—ROM, velocity, acceleration, and lumbopelvic rhythm (LPR)—across the trunk, thoracic, lumbar spine, and hip regions during both single‐plane and multi‐plane movements. LPR refers to the coordinated movement pattern between the lumbar spine and pelvis during activities such as bending, lifting, or twisting, as described in detail in the *Lumbopelvic Rhythm* section of Materials and Methods below. LPR plays a key role in overall spinal function, as the lumbar spine and pelvis must work together to facilitate movement. Although less commonly measured than ROM or velocity, LPR and acceleration may help identify compensatory mechanisms and altered musculoskeletal impairment related to cLBP [28, 29]. This novel approach enables a more thorough understanding of the complex biomechanical patterns associated with cLBP.

The University of Pittsburgh's Mechanistic Research Center (MRC) entitled, “Low Back Pain: Biological, Biomechanical, Behavioral Phenotypes (LB^3^P),” is a member of the National Institutes of Health's (NIH) Back Pain Consortium (BACPAC) Research Program—which is part of the Helping to End Addiction Long‐term (HEAL) Initiative. The overall objective of LB^3^P is to perform in‐depth phenotyping of patients with cLBP using a multi‐modal assessment approach that can inform improved treatments. The LB^3^P performed an observational cohort study of over 1000 people with cLBP [30]. This manuscript details the spinal and hip kinematics data from this cLBP cohort as part of a 9‐paper supplement aimed at reporting domain‐specific baseline data. By characterizing spinal and hip kinematics during standardized kinematic assessment and functional tasks, this study aims to provide a more nuanced understanding of the movement patterns in individuals with cLBP within a controlled (laboratory) setting.

## 2 Materials and Methods

LB^3^P conducted a prospective, longitudinal study with data collected from 1007 enrolled participants with cLBP. Descriptions of the data collection and processing of the biomechanical data were previously published by the BACPAC Biomechanic Working Group [31].

### 2.1 Participants

This study received Institutional Review Board (IRB) approval from the University of Pittsburgh. Complete descriptions of participant demographics and biomedical characteristics are described in detail elsewhere [32]. Inclusion criteria were English‐speaking adults who experienced cLBP, defined as pain between the lower posterior margin of the rib cage and the horizontal gluteal fold that persisted for at least 3 months and resulted in pain on at least half the days in the past 6 months [33]. Participants were excluded if they: (1) were not identified in the University of Pittsburgh Medical Center (UPMC) Electronic Health Record system, (2) were participating in a masked intervention study for LBP, or (3) had a medical condition that would place the participant at increased risk or preclude them from complying with study procedures. Participants were enrolled by clinician referral, research registries, and community announcements between June 2020 and March 2024. The in‐person enrollment visit took place at the University of Pittsburgh Department of Physical Therapy—Clinical and Translational Research Center. Participants were followed remotely for 12 months and were compensated for their participation incrementally at all timepoints [30, 34]; Data presented herein were collected at enrollment. This study was monitored by an independent observational study monitoring board and an advisory panel. This report follows the STrengthening the Reporting of OBservational Studies in Epidemiology (STROBE) reporting guidelines.

### 2.2 Instrumentation

#### 2.2.1 Wearable Motion Sensors

The wearable sensors and testing procedures used in this study were previously validated, demonstrating good‐to‐excellent concurrent validity between IMUs and optical motion capture systems and generally good‐to‐excellent repeated‐measures reliability for thoracic, lumbar, and hip ROM in the primary cardinal planes [35]. The concurrent validity and repeated‐measures reliability findings are consistent with other wearable IMU‐based systems used to measure human movement [36, 37, 38, 39]. These procedures were described in detail elsewhere [35, 40].

**FIGURE 1:**
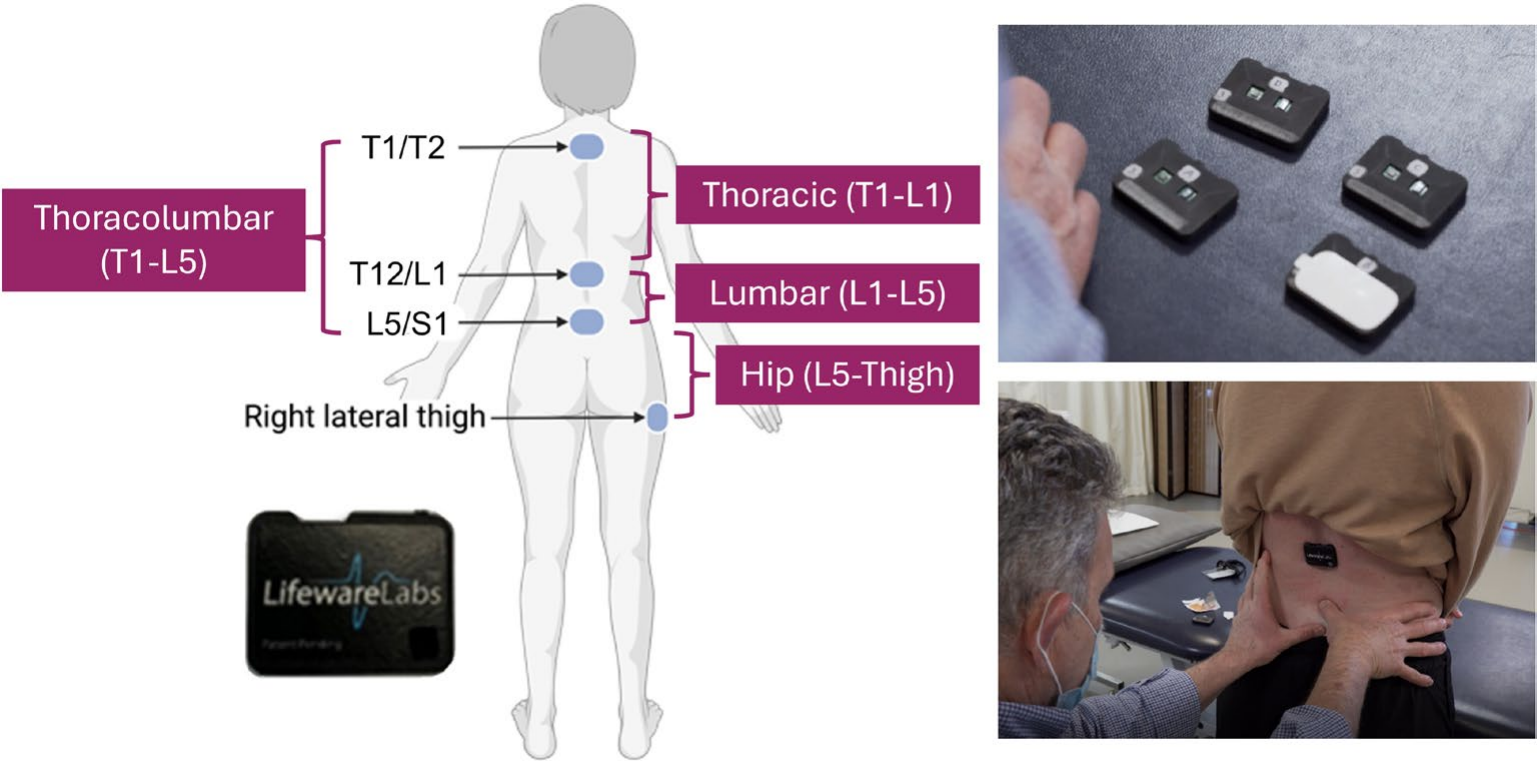
The left most image displays the locations of inertial measurement unit (IMU) sensors on the body during in‐clinic testing. Sensors were positioned at the following locations: T1/T2, T12/L1, L5/S1, and right lateral thigh and display the IMU segments. The two images to the right are still images illustrating the preparation and placement of IMUs. Top: Four IMUs are powered on and placed on a flat surface as the physical therapist applies skin adhesive to each unit (skin adhesive in white on lower right sensor). Bottom: The physical therapist palpates the L5/S1 anatomical landmark to ensure accurate placement of the sensors, while the T12/S1 sensor is already affixed on the skin over the spine.

Four inertial measurement units (IMU) were placed on the skin using double‐sided adhesive over the T1/T2, T12/L1, and L5/S1 interspinous places and along the right femur, 10 cm distal to the greater trochanter prominence along an imaginary line connecting the greater trochanter to the fibular head (Figure 1). The wearable sensors contained the BNO055 IMU (BOSCH Sensortec, Reutlingen, Germany) that includes a triple‐axis accelerometer (range of ±4G), triple‐axis gyroscope (range of ±250°/s), and triple‐axis magnetometer (ranges of ±1300 μT on the *x* and *y* axes and ±2500 μT on the *z* axis). Sensor orientation values in the form of quaternions were calculated with on‐board sensor fusion algorithms and transmitted along with raw sensor values. All sensor data were streamed to a local Android device (Pixel 3, Google LLC, Mountain View, CA) at a sampling rate of 62.5 Hz through a custom mobile application (Lifeware Labs LLC, Pittsburgh, PA), used for data capture, visualization of the data stream, and sensor calibration.

### 2.3 Testing Procedures

**FIGURE 2:**
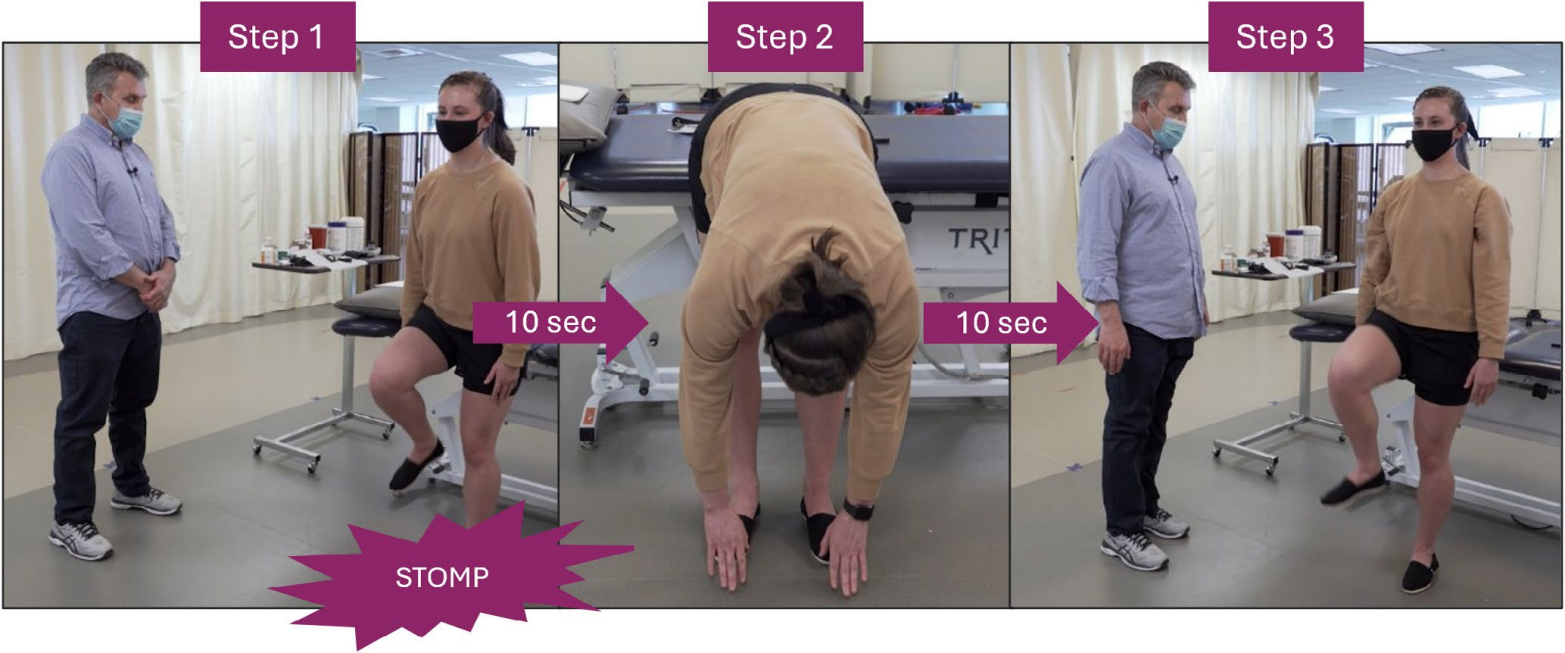
Three still images demonstrating the sensor synchronization procedure once all four sensors are placed on the participant's body. The physical therapist guides the participant through each motion. Left (Step 1): The participant remains upright and performs a stomp with their right leg to generate a spike in all four sensor data streams. Center (Step 2): The participant performs a forward bend to identify the horizontal axis in the frontal plane for the spinal sensors. Right (Step 3): The participant lifts their right thigh to identify the horizontal axis for the hip sensor. Each step is separated by a 10 s interval of quiet standing to find the vertical body axis and ensure clear signal differentiation.

**FIGURE 3:**
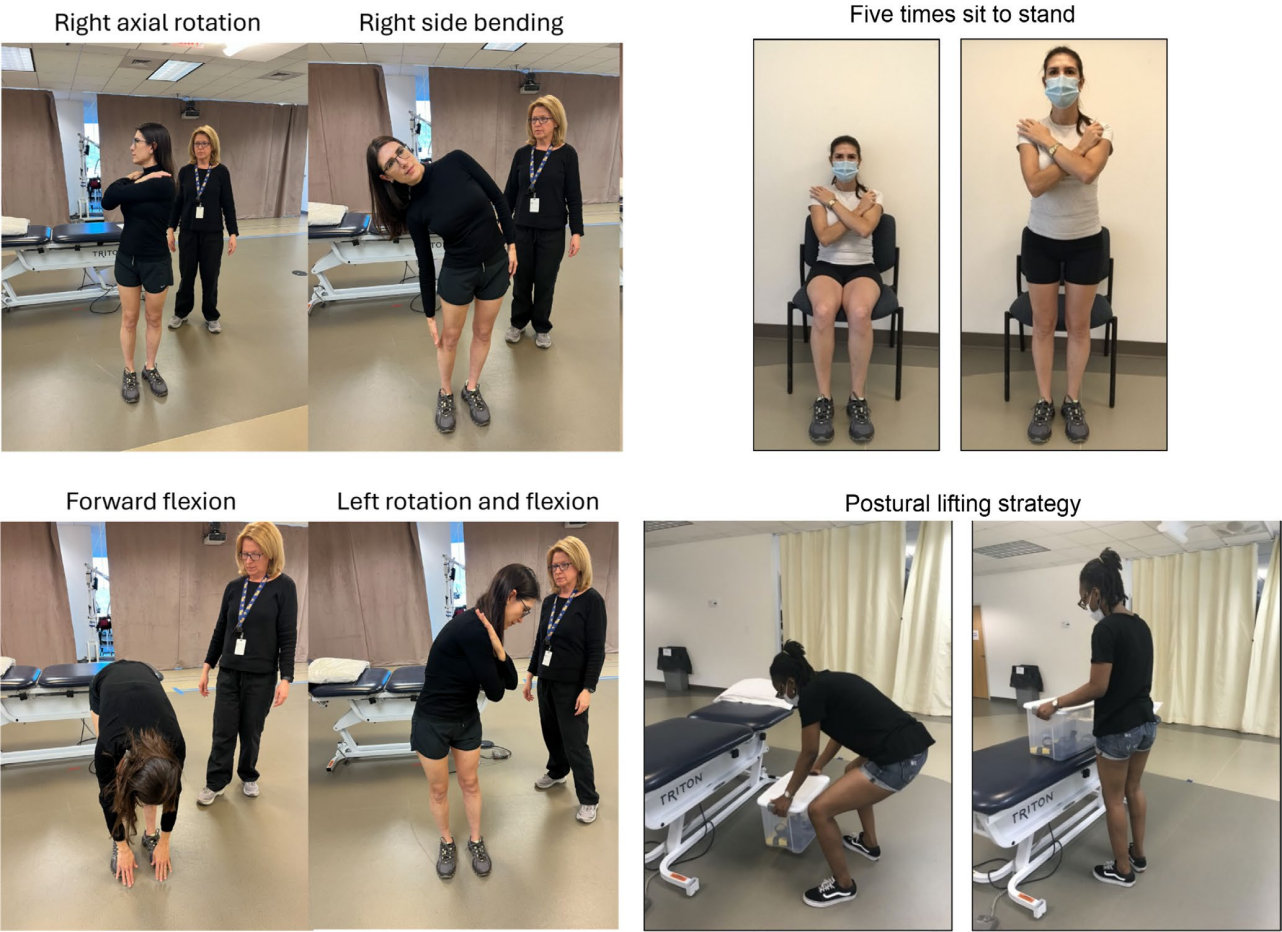
These images illustrate the physical assessments conducted under the supervision of a physical therapist. The leftmost grouping displays four ranges of motion (ROM) tests: (A) right axial rotation, (B) right lateral bending, (C) forward flexion, and (D) combined left rotation‐flexion, with the therapist present to ensure proper form and safety. The top right image shows two stages of the five times sit‐to‐stand (5STS) test, capturing the participant transitioning from a seated to a fully upright posture with arms crossed. The bottom right image depicts the postural lifting strategy (PLS) test, where the participant lifts a 3.6 kg box from the floor to a 75 cm high table using their preferred strategy.

Prior to placing the sensors, the physical therapists activated and performed the sensor calibration procedure. Calibration involved stacking all four sensors, placing them in one hand, and waving them in a “figure eight” to adjust the accelerometer, magnetometer, and gyroscope readings (Figure 2). The physical therapist palpated the spinous processes and greater trochanter, then cleaned the overlying skin using a SKIN‐PREP wipe (Smith & Nephew, Hull, England) before placing four IMUs along the participant's spine and right hip. The physical therapist then guided the participant through a sensor synchronization and anatomical alignment procedure involving a stomp, forward trunk bend, and right thigh lift with 10 s of quiet standing gaps in between (Figure 3). The stomp created a spike in the accelerometer data that was used to synchronize and initiate all four IMU data streams. The 10 s gap where the participant stood in a neutral standing position was used to find the vertical body axis, and the forward bend and thigh lift were used to find the horizontal axis in the frontal plane for the spinal and hip sensors, respectively. The third axis was found by taking the cross product, and a final cross product was taken to ensure orthogonality. To support standardization, all participants followed a predefined battery of exercises prompted by the mobile application, which included instructions and order of execution. Physical therapists administering the test were also instructed to follow this sequence to reduce potential variability due to task ordering. Participants were coached through tests and then instructed to complete each test on their own.

Kinematics were calculated for four regions—trunk (T1/L5), thoracic (T1/L1), lumbar (L1/L5), and hip (hip/L5)—across nine movements: self‐paced ROM—(1) axial rotation (AR), (2) lateral bending (LB), (3) flexion and extension (F/E); and fast‐paced ROM—(4) AR, (5) LB, (6) flexion, (7) coupled rotation/flexion (CRF), (8) five times sit‐to‐stand (5STS), and (9) postural lifting strategy (PLS). Metrics extracted from the motion data include ROM, angular velocity, angular acceleration, and lumbopelvic rhythm (LPR) (Table 1).

**TABLE 1:** Metrics collected across self‐paced and fast‐paced tests.

	Self‐paced tests			Fast‐paced tests					
	Axial rotation	Lateral bending	Flexion and extension	Axial rotation	Lateral bending	Flexion	Coupled rotation/flexion	Five times sit‐to‐stand	Postural lifting strategy
ROM	X	X	X				X	X	X
Velocity				X	X	X	X	X	X
Acceleration				X	X	X	X	X	X
LPR			X						

**FIGURE 4:**
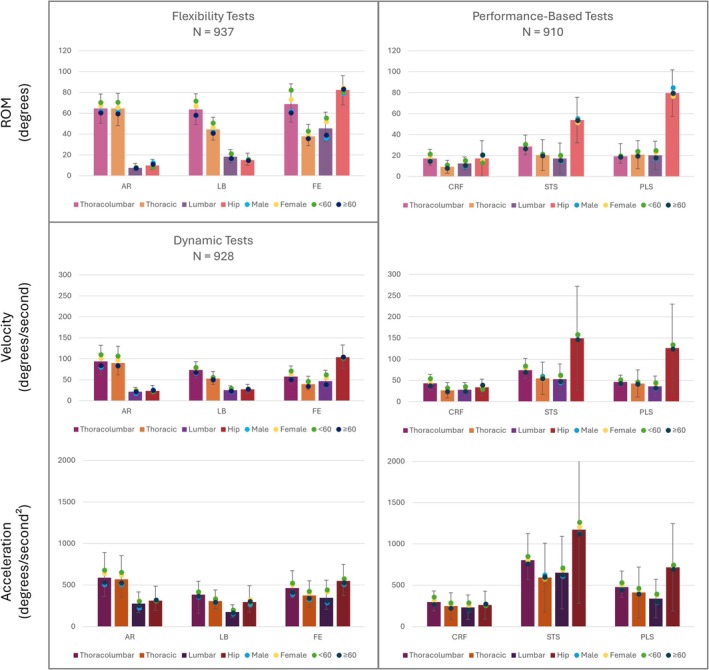
These bar graphs illustrate the results of the flexibility (self‐paced), dynamic (fast‐paced), and performance‐based tests with error bars for standard deviation and symbols for the sex and age‐based divisions. The top left graph is total ROM for flexibility tests; the top right is average ROM for performance‐based tests. The middle graphs are average velocity for dynamic tests (left) and performance‐based tests (right). The bottom graphs are average acceleration for dynamic tests (left) and performance‐based tests (right).

Participants were instructed to perform the movement to the left side first, return to center, and then move to the right side for bilateral tests. For self‐paced tests, participants were instructed to perform one repetition to each side and move *as far as* they could comfortably go. For fast‐paced tests, participants were coached to move *as fast as* they could comfortably go, completing three reps of AR (bilateral), LB (bilateral), flexion, and CRF (bilateral) (Figure 4). For CRF, participants were instructed to axially rotate their trunk as far as possible and hold that rotation while performing three forward bends from the waist. Across all standing tests, participants were instructed to keep their pelvis facing forward, knees straight, and isolate movement to the trunk. For 5STS, participants were instructed to cross their arms over their chest and move *quickly* between a full stand and full sit position five times (Figure 4). For PLS, participants were asked to lift a weighted box from the floor up to a 75‐cm high table and back [41] to the floor in a continuous motion for four repetitions or 20 s, whichever came first. Female participants used a 3.6 kg (~8 lb.) box, while male participants used a 5.85 kg (~13 lb.) box (Figure 4).

### 2.4 Data Collection and Processing

All kinematic data processing was completed using MATLAB R2022a (MathWorks Inc., Natick, MA). IMU‐to‐anatomical rotation matrices were calculated to account for the offset between the IMU and anatomic coordinate systems. These procedures and the associated analysis code are described comprehensively by Bailes et al. [35] Raw quaternion data calculated by the on‐board sensor fusion algorithm were converted into 3 × 3 rotation matrices, indicating rotation from the local IMU sensor coordinate system to the global reference frame for each time point. These rotation matrices were multiplied by the rotation matrix obtained from the alignment procedure to get the anatomical‐to‐global rotation matrices for each sensor (T1, L1, L5, and hip). Matrix multiplication was used to determine the relative positioning of one sensor in reference to another at each time point (Equation 1). To establish a neutral position for each participant's starting posture, a rotation matrix was created based on the average position from the first five index points of each trial. A sagittal/frontal/transverse decomposition order (F/E, LB, AR) was applied to calculate the Euler angles, where Equation (2) shows how any rotation matrix *R* can be decomposed into sequential rotations about the *Y*, *Z*, and *X* axes, represented by angles *β*, *γ*, and *α* respectively. The decomposition sequence ensures that these angles represent clinically meaningful motions by isolating the rotations for each axis in the specified order and minimize the chance of gimbal lock. Euler angles were unwrapped to correct discontinuities greater than π radians between consecutive angles, converted from radians to degrees, and filtered. The data were processed using a zero‐phase, third‐order Butterworth low‐pass filter with a sampling frequency of 62.5 Hz and a cutoff frequency of 5 Hz, which was shown to maximize the signal‐to‐noise ratio during preliminary testing.

(1) $$R_{L1\;\text{anatomical}}^{L5\;\text{anatomical}}=R_{L5\;\text{local}}^{L5\;\text{anatomical}}\cdot R_{\text{global}}^{L5\;\text{local}}\cdot R_{L1\;\text{local}}^{\text{global}}\cdot R_{L1\;\text{anatomical}}^{L1\;\text{local}}$$

(2) $$R=X\left(\alpha \right)Z\left(\gamma \right)Y\left(\beta \right)$$

Angular velocity in the IMU frame was calculated directly from raw gyroscope data rather than quaternions. The raw gyroscope data were filtered using the same low‐pass Butterworth filter and then multiplied by the rotation matrices to obtain anatomical‐frame velocity. Anatomical‐frame velocity data were differentiated with respect to time to calculate acceleration.

Maximum ROM in both directions was calculated, as well as total (overall) ROM for each self‐paced test (AR, LB, F/E). Trimmed means of velocity and acceleration were calculated for fast‐paced tests (AR, LB, flexion). ROM, velocity, and acceleration were calculated for fast‐paced tests (CRF, 5STS, PLS).

ROM, velocity, and acceleration data were plotted versus time, and the plots were visually inspected (RER), with additional consultation from the primary authors (KMB, WWC) when the kinematic trace deviated from the expected movement pattern. For each repetition, maximum and minimum peaks were extracted, and a trimmed mean of the absolute values of these peaks was used to calculate overall velocity and acceleration to reduce the impact of noise on the results. ROM for CRF, 5STS, and PLS was calculated based on excursion in the sagittal plane for each repetition. Movement peaks were converted to absolute values and averaged to calculate overall ROM.

Expected movement was defined as excursion, velocity, and acceleration within physiological ranges published in the literature, symmetrical data for bilateral movements, and movement following the global direction that was cued. In cases where unexpected movement patterns were not able to be resolved with the standard processing procedures, ROM was calculated using cumulative trapezoidal numerical integration of raw gyroscope data (integrated ROM). No data were excluded from the analysis, and outliers were included in the overall summary.

### 2.5 Lumbopelvic Rhythm

LPR refers to the coordinated interactions between the lumbar spine and hip during forward flexion and backward extension, with typical metrics including the relative magnitude of lumbar and hip contributions and the temporal sequence of their activation. To analyze LPR, the start and end of forward flexion and backward extension were manually selected through visual inspection of the self‐paced F/E data for the lumbar and hip regions.

To prevent the LPR ratio from approaching infinity when hip movement was minimal (0°–1°), the start of motion was defined based on the approach described by Tojima et al. [42], delaying the onset until hip ROM was ≥ 1°. Similarly, the end of movement was determined following Marich et al. [29], marking the completion of flexion or extension at 98% of the maximum trunk movement, depending on which region reached its maximum excursion later [29, 42]. Once start and stop were calculated, the ROM data were segmented into four equal quartiles (25%, 50%, 75%, and 100%) based on the time taken to get from when hip excursion surpassed 1° to 98% of the maximum movement. Then, the LPR ratio data were averaged across each quartile for neutral‐to‐flexion and neutral‐to‐extension to analyze the magnitude of each region's contribution to trunk motion.

### 2.6 Data Analysis

The metrics extracted from the motion data were:

1. Range of Motion (ROM) (°): Measured for the trunk (T1/L5), thoracic (T1/L1), lumbar (L1/L5), and hip (hip/L5) regions during self‐paced tests (AR, LB, F/E) and fast‐paced tests (CRF, 5STS, and PLS).
2. Angular Velocity (°/s): Measured for the trunk, thoracic, lumbar, and hip regions during fast‐paced tests (AR, LB, Flexion, CRF, 5STS, PLS).
3. Angular Acceleration (°/s^2^): Measured for the trunk, thoracic, lumbar, and hip regions during fast‐paced tests (AR, LB, Flexion, CRF, 5STS, PLS).
4. LPR Ratios: Calculated for each quartile of neutral‐to‐flexion and neutral‐to‐extension during self‐paced F/E.

The reported data represent descriptives for these metrics, collected during the participants' enrollment visits. Due to the large sample size, Kolmogorov–Smirnov tests for normality (*p* > 0.05) were conducted for the total cohort using SPSS v30 (IBM, Chicago, IL). The majority of variables exhibited a non‐normal distribution; therefore, medians and interquartile ranges (IQR) were calculated. Descriptive statistics are presented both overall and stratified by biological sex at birth (male, female) and age (≥ 60 years old, < 60 years old). 60 years old was the age selected for division of age groups based on a previous systematic review and meta‐analysis, which reported that individuals over the age of 60 experience a significantly higher prevalence of cLBP, distinct psychosocial challenges, and more varied pain experiences. These factors all support the need for more targeted management strategies in this population [43]. One participant reported intersex at birth and was therefore not included in the male/female comparison.

## 3 Results

A total of 1007 participants with cLBP were enrolled in the study. Of this cohort, 46 participants were screened out of in‐lab testing for the following reasons: withdrawal (1), refusal to proceed with further tests (2), presence of medical devices that precluded testing (4), severe pain (6), skin conditions preventing the use of adhesives (6), extreme blood pressure (8), and safety concerns (19). Additionally, data from seven participants were rendered unusable due to sensor streaming and resampling issues. In total, 954 participants had usable sensor data (Table 2). However, sample sizes varied across tests and segments due to partial test completion and sensor malfunctions. The sample size for each metric is listed in corresponding tables.

**TABLE 2:** Participant demographics (*N* = 954).

Age in years (Mean ± SD)	58.6 ± 16.4
Sex, *N* (%)	Male: 384 (40.3%) Female: 569 (59.6%) Intersex: 1 (0.1%)
BMI (Mean ± SD)	31.4 ± 7.4
Race, *N* (%)	White: 724 (75.9%) Black or African American: 166 (17.4%) Multiracial: 24 (2.5%) Asian: 19 (2.0%) Other: 4 (0.4%) Unknown/undisclosed: 17 (1.8%)
Osteoarthritis, *N* (%)	Knee: 165 (17%) Hip: 299 (31%)

### 3.1 Range of Motion

The total trunk, thoracic, lumbar, and hip excursion for the overall group, males, females, younger adults, and older adults during self‐paced AR, LB, F/E, and fast‐paced CRF, 5STS, and PLS is summarized in Tables 3, 4, 5, 6. The results highlight distinct patterns in ROM across different spinal and hip regions, depending on the type of movement evaluated. During isolated movements such as AR and LB, the trunk region demonstrated the highest ROM, while the hip region contributed the most to total ROM during F/E. In contrast, during functional tasks such as CRF, 5STS, and PLS, ROM was notably reduced across all spinal regions, with the hip region primarily having the largest excursion.

**TABLE 3:** Flexibility ROM: Axial rotation (median (IQR) in degrees).

		Axial rotation											
		T1/L5			T1/L1			L1/L5			Hip/L5		
		Left ROM	Right ROM	Total ROM	Left ROM	Right ROM	Total ROM	Left ROM	Right ROM	Total ROM	Left ROM	Right ROM	Total ROM
Total	(*N* = 936)	−32.4 (−39.4 to −25.0)*	32.4 (23.8–39.2)	64.7 (50.3–78.5)	−31.6 (−39.6 to −23.9)*	32.3 (23.3–40.3)	64.7 (48.1–79.0)*	−1.0 (−4.0–3.0)	1.3 (−3.8–4.3)	7.6 (5.1–12.0)	3.7 (1.5–6.9)	−5.5 (−9.7 to −2.3)	10.0 (6.0–15.8)
Male	(*N* = 375)	−31.2 (−37.9 to −22.0)	30.9 (20.7–38.6)	62.1 (43.1–74.6)	−31.0 (−39.1 to −22.1)	31.6 (20.8–40.0)	62.4 (42.7–77.6)	−0.02 (−2.8–3.5)	0.3 (−3.9–3.3)	7.2 (4.7–10.3)	4.8 (1.9–8.5)	−6.9 (−12.0 to −3.4)	12.7 (7.5–19.6)
Female	(*N* = 560)	−33.1 (−40.5 to −26.7)	33.7 (26.2–39.8)	67.2 (54.1–79.8)	−31.9 (−39.8 to −24.4)	32.9 (25.5–40.3)	65.5 (50.8–80.0)	−1.6 (−4.7–2.5)	1.9 (−3.6–5.1)	8.2 (5.6–12.7)	3.3 (1.3–5.9)	−4.6 (−8.5 to −1.8)	8.8 (5.5–13.6)
≥ 60 years old	(*N* = 534)	−30.4 (−37.5 to −22.8)	30.2 (21.4–37.3)	60.6 (45.1–74.0)	−29.9 (−37.3 to −21.9)	29.9 (21.2–37.3)	59.5 (44.8–74.1)	−1.0 (−3.8–2.9)	1.3 (−3.4–4.2)	7.4 (4.9–12.0)	4.2 (1.8–7.4)	−6.4 (−11.8 to −3.3)	11.1 (7.1–18.1)
< 60 years old	(*N* = 402)	−35.2 (−41.2 to −27.9)	35.1 (27.8–42.0)	70.4 (57.7–83.7)	−34.2 (−41.6 to −26.7)	35.5 (27.1–43.3)	70.6 (56.2–84.7)	−1.0 (−4.3–3.1)	1.3 (−4.2–4.6)	8.1 (5.6–12.0)	3.4 (1.2–6.0)	−4.2 (−8.0 to −1.6)	8.3 (5.4–13.7)

* Indicates variables that were normally distributed for the total cohort based on the Kolmogorov–Smirnov test (*p* > 0.05).

**TABLE 4:** Flexibility ROM: Lateral bending (median (IQR) in degrees).

		Lateral bend											
		T1/L5			T1/L1			L1/L5			Hip/L5		
		Left ROM	Right ROM	Total ROM	Left ROM	Right ROM	Total ROM	Left ROM	Right ROM	Total ROM	Left ROM	Right ROM	Total ROM
Total	(*N* = 937)	33.2 (25.3–40.7)*	−30.4 (−38.0 to −22.9)*	63.8 (49.2–78.7)*	23.4 (18.2–29.6)	−21.0 (−27.8 to −16.0)	44.6 (34.3–56.1)*	9.3 (6.0–13.2)	−9.0 (−12.4 to −5.7)	18.3 (12.6–25.2)	−6.6 (−10.1 to −4.2)	8.2 (5.1–12.3)	15.0 (10.2–21.7)
Male	(*N* = 376)	30.1 (22.2–37.7)	−27.7 (−35.5 to −20.7)	57.8 (43.2–71.5)	22.4 (16.8–27.1)	−19.6 (−25.8 to −14.9)	42.2 (32.1–52.8)	7.6 (4.9–10.9)	−7.8 (−11.1 to −4.8)	16.5 (10.8–21.6)	−7.2 (−10.7 to −4.5)	8.8 (5.5–12.7)	15.8 (10.3–22.9)
Female	(*N* = 560)	35.4 (27.7–43.3)	−32.0 (−39.1 to −24.8)	67.0 (53.7–81.8)	24.2 (19.4–31.0)	−22.2 (−28.5 to −16.6)	46.5 (36.5–58.3)	10.5 (6.9–14.2)	−9.7 (−13.3 to −6.2)	20.7 (14.3–26.8)	−6.3 (−9.6 to −4.1)	7.8 (5.1–11.8)	14.3 (10.1–20.8)
≥ 60 years old	(*N* = 535)	30.2 (23.2–37.2)	−27.4 (−34.6 to −21.3)	58.3 (45.2–70.1)	21.4 (16.8–26.7)	−19.5 (−25.2 to −14.9)	41.1 (32.5–51.9)	8.3 (5.0–12.4)	−7.6 (−11.3 to −4.4)	16.7 (10.7–22.9)	−6.4 (−9.7 to −4.1)	7.8 (5.1–12.0)	14.7 (10.1–20.9)
< 60 years old	(*N* = 402)	37.6 (28.9–43.7)	−34.6 (−41.9 to −26.8)	71.7 (58.2–85.4)	26.3 (20.2–31.6)	−24.1 (−30.3 to −17.7)	50.8 (39.1–61.8)	10.6 (6.9–14.3)	−10.5 (−14.0 to −7.1)	21.3 (15.5–27.4)	−7.0 (−10.5 to −4.4)	8.6 (5.5–12.6)	15.7 (10.6–22.4)

* Indicates variables that were normally distributed for the total cohort based on the Kolmogorov–Smirnov test (*p* > 0.05).

**TABLE 5:** Flexibility ROM: Flexion and extension (median (IQR) in degrees).

		Flexion and extension											
		T1/L5			T1/L1			L1/L5			Hip/L5		
		Flexion	Extension	Total ROM	Flexion	Extension	Total ROM	Flexion	Extension	Total ROM	Flexion	Extension	Total ROM
Total	(*N* = 931)	−36.5 (−50.8 to −25.4)	30.4 (20.4–42.3)	68.8 (51.5–88.3)	−15.4 (−23.1 to −8.9)	20.8 (13.2–30.2)	37.9 (29.0–49.4)	−28.4 (−39.7 to −18.8)	16.0 (9.8–23.8)	45.6 (32.7–61.2)	67.9 (53.7–81.4)*	−14.3 (−19.6 to −9.0)	82.4 (68.0–96.2)*
Male	(*N* = 372)	−32.9 (−43.0 to −20.5)	27.3 (18.2–38.9)	61.9 (43.5–79.4)	−15.8 (−23.5 to −8.6)	19.5 (12.7–28.6)	37.6 (27.8–48.1)	−21.9 (−30.0 to −15.1)	13.0 (8.1–19.3)	36.2 (27.6–47.5)	67.3 (53.7–81.1)	−12.4 (−17.5 to −8.4)	79.2 (66.5–94.4)
Female	(*N* = 558)	−40.6 (−56.1 to −28.2)	32.7 (22.3–43.4)	73.3 (57.6–92.8)	−15.1 (−22.8 to −9.1)	21.4 (13.7–30.7)	38.3 (29.2–50.5)	−33.6 (−45.2 to −22.4)	17.5 (11.2–26.6)	51.8 (39.2–67.9)	68.5 (53.4–81.5)	−15.5 (−20.8 to −9.9)	84.4 (69.4–97.6)
≥ 60 years old	(*N* = 532)	−32.3 (−42.8 to −20.5)	27.0 (18.2–36.3)	60.5 (45.1–76.4)	−14.8 (−21.5 to −8.2)	19.0 (11.8–27.4)	35.8 (26.9–46.2)	−22.7 (−33.0 to −15.6)	13.5 (8.1–20.0)	39.0 (28.8–51.7)	70.3 (55.3–81.2)	−13.9 (−19.1 to −9.3)	83.3 (71.3–96.1)
< 60 years old	(*N* = 399)	−44.2 (−59.3 to −31.3)	36.9 (24.3–50.2)	82.3 (64.5–101.9)	−16.1 (−24.8 to −10.1)	23.7 (15.9–33.9)	43.0 (31.9–53.7)	−35.4 (−48.6 to −25.9)	19.4 (12.3–27.7)	55.6 (40.8–72.7)	64.9 (50.3–81.6)	−14.7 (−20.5 to −8.9)	80.7 (65.6–96.1)

* Indicates variables that were normally distributed for the total cohort based on the Kolmogorov–Smirnov test (*p* > 0.05).

**TABLE 6:** Dynamic motion: Flexion/range of motion (median (IQR) in degrees).

		Coupled rotation/flexion				Five times sit to stand				Postural lifting strategy			
		T1/L5	T1/L1	L1/L5	Hip/L5	T1/L5	T1/L1	L1/L5	Hip/L5	T1/L5	T1/L1	L1/L5	Hip/L5
Total	(*N* = 909)	17.2 (10.6–25.9)	9.1 (6.2–13.4)	12.5 (6.8–20.0)	17.2 (9.4–27.6)	28.5 (20.7–39.6)	20.4 (14.7–27.3)	17.2 (10.7–26.6)	53.8 (43.4–65.0)	19.3 (12.7–31.4)	20.8 (13.5–31.9)	20.2 (11.6–31.6)	79.5 (61.3–94.0)
Male	(*N* = 369)	15.7 (9.5–23.2)	8.8 (5.9–13.1)	10.1 (5.9–16.4)	20.3 (11.8–31.9)	29.3 (20.6–39.7)	21.4 (15.9–28.0)	14.7 (9.2–22.3)	55.3 (46.0–65.8)	18.7 (12.3–30.6)	19.8 (12.7–30.7)	17.0 (9.2–24.3)	84.9 (68.4–97.6)
Female	(*N* = 539)	18.4 (12.0–27.4)	9.4 (6.4–13.6)	13.9 (7.7–22.2)	15.3 (8.8–24.7)	28.2 (20.8–39.3)	19.6 (14.1–26.9)	19.3 (12.6–28.8)	51.5 (41.6–63.8)	19.7 (13.1–31.6)	22.2 (14.0–33.3)	23.2 (14.5–34.6)	76.7 (59.0–91.0)
≥ 60 years old	(*N* = 520)	14.7 (9.4–21.7)	8.0 (5.4–11.9)	10.5 (5.9–16.5)	20.7 (12.0–31.4)	26.6 (19.4–36.7)	19.7 (14.3–26.7)	15.3 (9.6–22.7)	53.5 (43.3–64.7)	18.7 (12.4–30.0)	19.5 (12.4–28.9)	17.9 (10.0–26.2)	79.7 (63.4–93.3)
< 60 years old	(*N* = 389)	21.5 (14.0–31.3)	11.1 (7.6–15.0)	15.4 (9.1–25.3)	13.1 (7.7–20.6)	30.8 (23.3–42.0)	21.4 (15.4–28.6)	20.4 (13.3–29.9)	53.9 (43.6–65.8)	19.8 (13.2–33.1)	24.2 (14.6–36.0)	24.9 (15.1–36.8)	79.2 (59.5–94.9)

The data revealed differing degrees of variability within the ROM metric across different movement types when comparing median values to the IQRs (Tables 3, 4, 5, 6). The lumbar region demonstrated considerable variability during AR, with wide IQRs for left and right ROM relative to the median, likely due to lumbar excursion opposite the direction cued (not due to a misunderstanding of directions, but due to differences in individual movement strategies and spine mobility constraints). In contrast, the thoracic region exhibited proportionally lower variability relative to its median values during both AR and LB. When bilateral measurements were combined, the total AR lumbar range showed more moderate variability. For AR, the relatively low thoracic variability contrasted with higher variability in the lumbar and hip regions. LB followed a similar pattern, with lower proportional variability in the thoracic region but wider ranges in the lumbar and hip segments relative to their median values. During FE, the thoracic spine demonstrated the greatest proportional variability, particularly in the flexion phase. The dynamic tasks (CRF, 5STS, and PLS) showed consistently higher relative variability in the lumbar region compared to other segments.

Sex and age analyses revealed additional insights into variability in ROM. Female participants consistently exhibited greater ROM compared to males, particularly at the trunk region during AR and LB, and at the hip region during F/E. However, exceptions emerged, especially at the hip segment, where sex and age influenced movement patterns differently. In general, older participants (≥ 60 years old) demonstrated reduced spinal flexibility compared to younger participants (< 60 years old), most notably in AR and LB in the trunk segment. This pattern reversed during F/E at the hip segment, where older participants showed greater hip ROM.

### 3.2 Velocity and Acceleration

Velocity and acceleration results for fast‐paced tests (AR, LB, flexion, CRF, 5STS, PLS) are summarized in Tables 7, 8, 9, 10. During fast‐paced tests, including AR, LB, and flexion, participants consistently demonstrated higher velocities and accelerations in the trunk region compared to lower spinal and hip regions. For example, during AR and LB, the trunk region exhibited the greatest velocities, while the hip region showed comparatively lower values. In contrast, functional tasks like PLS exhibited the highest velocities and accelerations at the hip segment.

**TABLE 7:** Dynamic motion: Velocity (median (IQR) in degrees/s).

		Axial rotation				Lateral bend				Flexion			
		T1/L5	T1/L1	L1/L5	Hip/L5	T1/L5	T1/L1	L1/L5	Hip/L5	T1/L5	T1/L1	L1/L5	Hip/L5
Total	(*N* = 928)	93.9 (62.0–132.7)	90.0 (61.5–129.9)	21.6 (13.4–32.3)	23.5 (15.4–36.6)	73.2 (54.5–93.1)	52.6 (38.9–69.7)	25.1 (16.9–35.3)	27.3 (18.6–39.1)	57.7 (39.6–83.1)	39.8 (26.2–58.2)	46.7 (28.5–72.6)	103.6 (77.0–132.9)
Male	(*N* = 372)	79.8 (54.0–118.9)	82.9 (54.7–122.1)	17.6 (11.1–27.5)	25.4 (16.5–38.9)	66.2 (49.6–90.5)	49.4 (36.0–67.5)	21.7 (14.2–31.7)	26.7 (17.9–39.4)	51.1 (33.6–75.5)	34.4 (22.3–53.9)	37.1 (23.9–58.2)	102.2 (73.9–132.5)
Female	(*N* = 555)	100.6 (71.7–138.1)	96.9 (66.7–134.6)	24.5 (15.7–35.3)	22.0 (15.1–34.5)	75.7 (58.8–94.4)	53.6 (40.8–70.3)	26.5 (19.2–37.0)	27.5 (19.1–38.3)	62.9 (43.1–87.7)	42.5 (28.3–60.1)	54.7 (34.2–80.8)	104.6 (80.7–133.0)
≥ 60 years old	(*N* = 529)	83.5 (56.2–118.1)	83.0 (56.6–117.3)	20.6 (11.6–30.6)	24.7 (16.1–35.9)	67.5 (51.9–87.2)	49.8 (37.1–65.4)	22.4 (15.1–31.8)	26.9 (18.9–36.9)	50.3 (34.1–70.1)	34.2 (23.1–52.1)	37.7 (24.0–59.2)	104.0 (80.7–130.9)
< 60 years old	(*N* = 399)	109.6 (76.8–150.3)	106.3 (71.5–146.4)	23.7 (15.2–35.5)	21.9 (14.8–37.4)	79.1 (61.1–102.2)	55.7 (42.4–74.0)	29.1 (19.6–39.7)	27.7 (18.5–40.7)	70.5 (48.9–98.6)	46.0 (29.5–63.7)	61.9 (39.5–93.2)	101.7 (74.2–136.3)

**TABLE 8:** Dynamic motion: Velocity (median (IQR) in degrees/s).

		Coupled rotation/flexion				Five times sit to stand				Postural lifting strategy			
		T1/L5	T1/L1	L1/L5	Hip/L5	T1/L5	T1/L1	L1/L5	Hip/L5	T1/L5	T1/L1	L1/L5	Hip/L5
Total	(*N* = 909)	43.1 (26.4–64.2)	26.8 (18.0–40.3)	28.2 (16.7–43.6)	33.8 (19.6–53.1)	74.1 (54.8–101.7)	54.9 (38.3–75.5)	52.9 (36.0–80.5)	149.4 (122.5–181.2)	46.7 (35.6–62.6)	42.9 (31.8–59.4)	36.0 (24.2–54.9)	126.6 (103.7–157.0)
Male	(*N* = 369)	39.4 (23.3–61.0)	26.2 (16.3–40.5)	23.2 (13.5–36.2)	38.3 (22.1–58.6)	71.9 (52.9–99.2)	59.8 (39.2–79.6)	44.3 (33.3–73.8)	157.6 (124.4–185.6)	42.2 (33.9–58.3)	40.3 (29.5–52.5)	29.6 (20.1–43.8)	133.1 (107.9–162.2)
Female	(*N* = 539)	44.6 (28.9–65.7)	27.3 (18.7–40.2)	31.0 (19.0–46.6)	31.5 (18.7–49.7)	77.0 (55.3–103.4)	52.8 (37.6–72.5)	59.8 (39.8–83.0)	145.8 (122.2–175.0)	49.1 (36.5–66.2)	44.9 (33.0–60.7)	41.2 (27.8–61.1)	124.0 (100.1–152.9)
≥ 60 years old	(*N* = 520)	37.2 (22.5–52.7)	23.1 (15.4–35.3)	23.9 (14.0–35.4)	38.9 (23.2–59.6)	69.5 (51.2–95.9)	53.7 (37.4–73.2)	46.7 (33.6–74.4)	146.1 (121.3–173.9)	42.2 (34.2–59.5)	40.6 (29.7–53.9)	31.4 (21.3–45.4)	123.6 (99.3–154.3)
< 60 years old	(*N* = 389)	54.1 (34.6–77.2)	31.6 (22.9–47.3)	34.9 (21.3–52.7)	29.1 (16.8–45.1)	83.6 (60.5–111.7)	56.4 (40.4–76.3)	61.6 (41.4–85.2)	158.2 (126.0–189.6)	51.2 (38.5–66.3)	45.7 (34.7–62.6)	44.6 (29.6–63.2)	133.4 (108.2–160.3)

**TABLE 9:** Dynamic motion: Acceleration (median (IQR) in degrees/second^2^ e + 02).

		Axial rotation				Lateral bend				Flexion			
		T1/L5	T1/L1	L1/L5	Hip/L5	T1/L5	T1/L1	L1/L5	Hip/L5	T1/L5	T1/L1	L1/L5	Hip/L5
Total	(*N* = 927)	5.9 (3.6–8.9)	5.7 (3.5–8.5)	2.8 (1.7–4.2)	3.1 (2.0–4.9)	3.8 (2.7–5.4)	3.1 (2.1–4.4)	1.8 (1.1–2.6)	3.0 (1.7–4.9)	4.6 (2.9–6.7)	3.8 (2.3–5.5)	3.5 (2.1–5.6)	5.5 (3.7–7.5)
Male	(*N* = 372)	5.0 (3.1–8.2)	5.0 (3.2–7.9)	2.2 (1.3–3.5)	3.1 (1.9–4.9)	3.5 (2.3–5.3)	2.9 (1.9–4.4)	1.5 (0.9–2.4)	2.6 (1.5–4.3)	3.8 (2.5–6.0)	3.2 (2.0–5.0)	2.9 (1.7–4.4)	5.1 (3.3–6.8)
Female	(*N* = 554)	6.4 (4.2–9.2)	6.1 (4.0–8.7)	3.1 (1.9–4.6)	3.1 (2.0–4.8)	4.0 (3.0–5.4)	3.1 (2.3–4.4)	1.9 (1.3–2.7)	3.2 (1.9–5.4)	5.0 (3.3–7.2)	4.0 (2.6–6.0)	4.2 (2.5–6.3)	5.7 (4.0–7.8)
≥ 60 years old	(*N* = 529)	5.3 (3.3–8.0)	5.2 (3.3–7.7)	2.5 (1.4–3.9)	3.2 (2.0–4.8)	3.6 (2.5–5.0)	2.9 (2.1–4.2)	1.6 (1.0–2.4)	3.0 (1.9–5.0)	4.1 (2.5–6.1)	3.4 (2.1–5.1)	3.0 (1.8–4.6)	5.3 (3.7–7.2)
< 60 years old	(*N* = 400)	6.8 (4.1–10.2)	6.5 (4.1–9.7)	3.0 (1.9–4.6)	3.0 (1.9–4.9)	4.2 (3.0–6.0)	3.3 (2.3–4.8)	2.0 (1.2–2.8)	2.9 (1.7–4.8)	5.2 (3.4–7.6)	4.2 (2.8–6.3)	4.4 (2.7–7.0)	5.7 (3.7–7.7)

**TABLE 10:** Dynamic motion: Acceleration (median (IQR) in degrees/second^2^ e + 02).

		Coupled rotation/flexion				Five times sit to stand				Postural lifting strategy			
		T1/L5	T1/L1	L1/L5	Hip/L5	T1/L5	T1/L1	L1/L5	Hip/L5	T1/L5	T1/L1	L1/L5	Hip/L5
Total	(*N* = 909)	3.0 (1.9–4.3)	2.5 (1.6–3.6)	2.3 (1.5–3.5)	2.6 (1.7–3.9)	8.0 (5.7–11.3)	5.9 (4.2–8.0)	6.5 (4.4–10.2)	11.7 (8.9–15.3)	4.8 (3.5–6.7)	4.1 (3.1–5.8)	3.4 (2.3–5.0)	7.2 (5.3–9.3)
Male	(*N* = 369)	2.7 (1.7–4.3)	2.3 (1.4–3.5)	2.0 (1.3–3.2)	2.6 (1.6–3.8)	7.9 (5.4–11.3)	6.2 (4.3–8.5)	6.1 (4.2–9.5)	11.2 (8.3–14.5)	4.5 (3.3–6.3)	4.1 (3.1–5.6)	2.9 (2.1–4.3)	7.3 (5.3–9.4)
Female	(*N* = 539)	3.1 (2.2–4.4)	2.6 (1.8–3.7)	2.6 (1.7–3.7)	2.6 (1.7–3.9)	8.1 (5.9–11.3)	5.7 (4.1–7.8)	6.8 (4.6–10.3)	12.0 (9.1–15.9)	5.0 (3.7–7.0)	4.1 (3.1–5.9)	3.8 (2.5–5.4)	7.1 (5.2–9.2)
≥ 60 years old	(*N* = 520)	2.7 (1.7–3.7)	2.2 (1.4–3.1)	2.0 (1.2–3.1)	2.7 (1.7–4.1)	7.6 (5.5–10.7)	6.0 (4.2–8.1)	6.2 (4.2–9.9)	11.2 (8.3–15.0)	4.4 (3.3–6.4)	3.9 (2.9–5.3)	3.0 (2.2–4.6)	6.9 (5.1–8.8)
< 60 years old	(*N* = 389)	3.6 (2.3–5.2)	2.9 (2.0–4.3)	2.9 (1.8–4.2)	2.5 (1.6–3.6)	8.5 (6.0–12.3)	5.9 (4.2–8.0)	7.1 (4.8–10.7)	12.6 (9.5–16.2)	5.3 (3.9–7.0)	4.6 (3.3–6.4)	3.9 (2.7–5.6)	7.4 (5.6–9.7)

Variability in velocity and acceleration metrics ranged from moderate to high across all tests (Tables 7, 8, 9, 10). For flexion, 5STS, and PLS, the lumbar region consistently exhibited large IQRs in velocity relative to median values, while the hip region showed more proportional variability, despite larger absolute ranges. LB demonstrated the most consistent velocity patterns with smaller IQRs across all regions. In terms of acceleration, PLS showed relatively uniform variability across regions, while 5STS exhibited notably high variability in the hip region. The trunk demonstrated consistently wider velocity ranges during AR, while maintaining moderate acceleration variability. The lumbar region, despite smaller absolute IQRs in acceleration, showed proportionally high variability relative to its median values, particularly during 5STS and CRF tasks.

Demographic patterns emphasized additional differences in movement dynamics. Female participants consistently exhibited higher velocities and accelerations compared to males across most tests, particularly in the trunk region during AR and LB. Similarly, younger participants (< 60 years old) demonstrated greater velocities and accelerations than older participants (≥ 60 years). These age‐related disparities were most pronounced during functional tasks. For example, during 5STS, younger participants showed more dynamic movement in their trunk compared to their older counterparts, and PLS revealed a similar trend at the hip segment.

### 3.3 Lumbopelvic Rhythm

**FIGURE 5:**
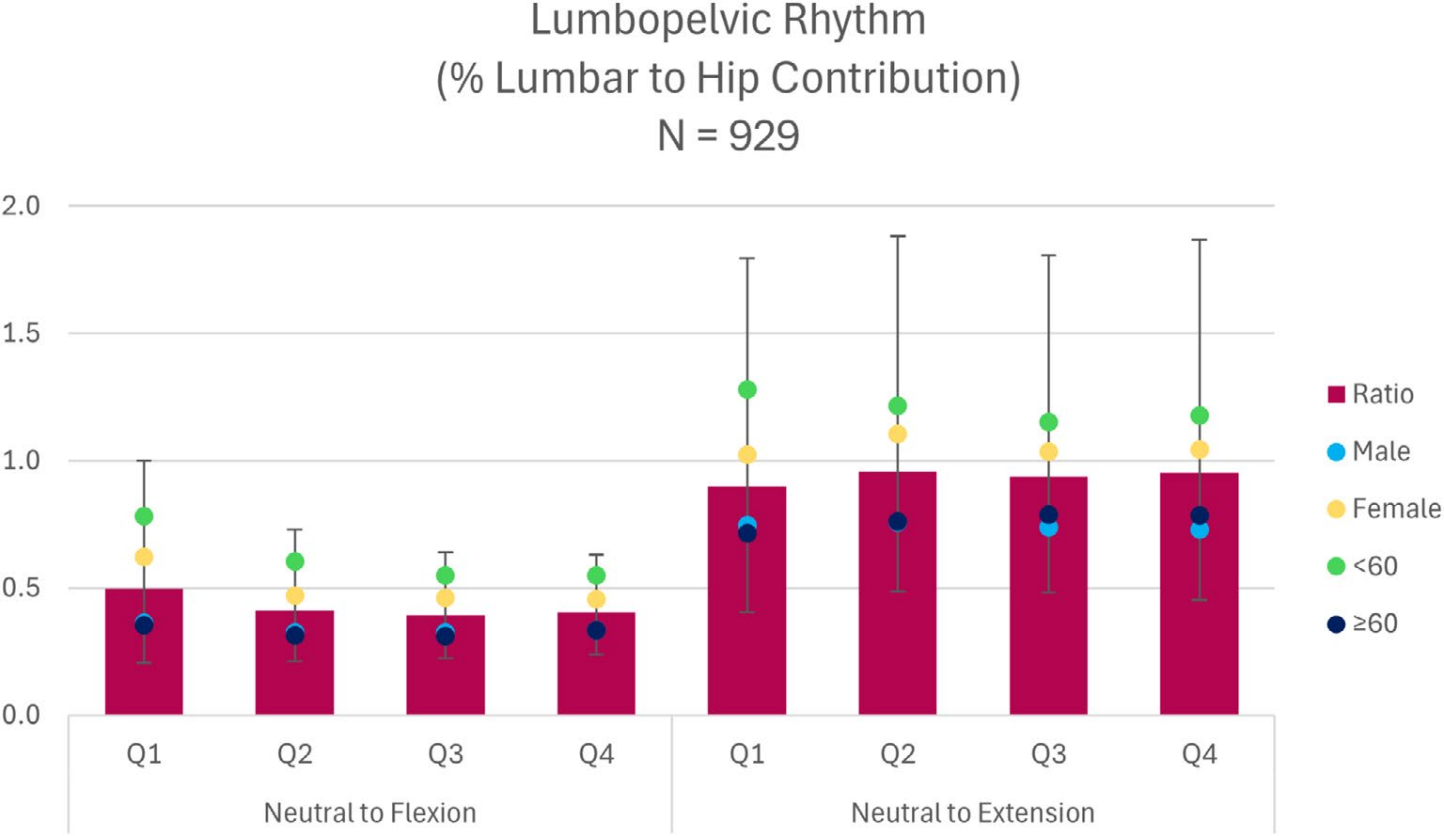
This graph displays the results for four quartiles (25%, 50%, 75%, and 100%) of lumbopelvic rhythm during neutral to maximal flexion and neutral to maximal extension.

Data for LPR during neutral‐to‐flexion and neutral‐to‐extension are presented in Table 11 and Figure 5. An LPR ratio greater than one indicates a greater contribution from the lumbar region, a ratio less than one indicates a greater contribution from the hip region, and a ratio of one signifies equal contributions from both regions.

**TABLE 11:** Lumbopelvic rhythm ratio.

		Neutral to flexion				Neutral to extension			
		Quartile 1	Quartile 2	Quartile 3	Quartile 4	Quartile 1	Quartile 2	Quartile 3	Quartile 4
Total	(*N* = 929)	0.5 (0.2–1.0)	0.4 (0.2–0.7)	0.4 (0.2–0.6)	0.4 (0.2–0.6)	0.9 (0.4–1.8)	1.0 (0.5–1.9)	0.9 (0.5–1.8)	1.0 (0.5–1.9)
Male	(*N* = 371)	0.4 (0.2–0.7)	0.3 (0.2–0.5)	0.3 (0.2–0.5)	0.3 (0.2–0.5)	0.7 (0.4–1.5)	0.8 (0.4–1.5)	0.7 (0.4–1.6)	0.7 (0.3–1.5)
Female	(*N* = 557)	0.6 (0.2–1.2)	0.5 (0.3–0.8)	0.5 (0.3–0.7)	0.5 (0.3–0.7)	1.0 (0.4–2.0)	1.1 (0.6–2.0)	1.0 (0.5–2.0)	1.0 (0.5–2.3)
≥ 60 years old	(*N* = 529)	0.4 (0.1–0.7)	0.3 (0.2–0.5)	0.3 (0.2–0.5)	0.3 (0.2–0.5)	0.7 (0.3–1.5)	0.8 (0.4–1.5)	0.8 (0.4–1.4)	0.8 (0.4–1.5)
< 60 years old	(*N* = 400)	0.8 (0.3–1.5)	0.6 (0.3–1.0)	0.5 (0.3–0.8)	0.6 (0.3–0.8)	1.3 (0.6–2.3)	1.2 (0.6–2.4)	1.2 (0.6–2.5)	1.2 (0.6–2.6)

LPR ratios revealed generally larger contributions from the pelvis during neutral to flexion for the total cohort and all demographic subgroups. For neutral to extension, the total cohort exhibited values close to or slightly below one. Male and ≥ 60 years old participants tended to have slightly lower LPR ratios compared to female and < 60 years old participants, indicating slightly larger hip excursion during forward flexion.

Differences in LPR for demographic groups were more pronounced during neutral to extension. Male and ≥ 60‐year‐old participants exhibited pelvic‐led movement across all quartiles of neutral to extension. In contrast, female participants had ratios that generally indicated equal contributions between segments, except during the second quartile, where the ratio slightly exceeded one, indicating greater lumbar excursion. Participants < 60 years old were the only group to demonstrate larger lumbar excursion than hip excursion during neutral to extension, with the highest ratios observed in the first quartile.

## 4 Discussion

The LB^3^P study was designed to collect a comprehensive and unbiased dataset from 1000 individuals to facilitate understanding of multiple contributors to pain and enable phenotyping of cLBP [30]. This paper focused on the biomechanical characteristics derived from data collected during the in‐person enrollment visit, specifically examining spinal and hip kinematics during a wide range of tasks (AR, LB, F/E, CRF, 5STS, PLS) using wearable motion sensors in a controlled clinical laboratory setting. Analyses included both overall data and stratifications based on biological sex (male, female) and age (≥ 60 years old, < 60 years old). The values established in this cohort provide a foundation for phenotyping subgroups of individuals with cLBP, offering insights to guide individualized treatment plans and inform clinical guidelines. Variations in kinematic data were observed based on body regions and movement types. Notably, the hip region played a critical role in functional tasks and LPR. Sex‐ and age‐based analyses unveiled consistent differences, with females generally exhibiting greater ROM and higher velocities compared to males, and younger participants (< 60 years old) also showing more dynamic movement patterns, except in the hip region during F/E, where older (≥ 60) participants exhibited greater excursion.

Overall, the data collection and data processing procedures implemented in this study resulted in excellent data quality. Expected movement patterns were observed in 97% of all participant movements, and the implementation of the integrated ROM approach to resolve the unexpected data further improved the overall data quality. The hypothesized cause of the unexpected movement patterns was insufficient calibration of the onboard sensor fusion algorithm. The integrated ROM approach was derived from the raw data (ignoring the sensor fusion algorithm output) and was therefore able to resolve the majority of unexpected movement data. Since no data were excluded, the wide interquartile ranges and higher medians observed may be attributable to the influence of potential outliers. When considering primary (cardinal plane) movements (F/E, LB, and AR), the mean lumbar ROM reported in the literature is highly variable, primarily due to methodological differences between studies (sensor type, sensor placement, regions captured, sample size, etc.). Reported ROM ranges collected by non‐invasive methods include: AR: 3°–62°; LB: 3°–44°; flexion: 23°–92°; extension: 15°–56° [17]. Although it is challenging to make direct comparisons, the findings of this study align with these patterns, with lumbar F/E exhibiting larger ROM than LB or AR, all within the reported ranges [17].

Similar sex‐ and age‐related lumbar ROM trends to those reported in the literature were observed. Arshad et al. noted variable sex‐based differences and generally greater lumbar ROM among participants under 60 years old compared to those 60 and older [44]. These age‐ and sex‐related differences in this study were consistent across other spinal regions, with females and younger participants (< 60 years old) displaying greater ROM. Female participants consistently exhibited greater ROM than males, aligning with prior findings that females tend to have greater excursion. However, this trend reversed for hip ROM, where older participants (≥ 60 years old) and males demonstrated greater excursion.

Functional tasks (CRF, 5STS, PLS) demonstrated lower spinal movement magnitudes compared to primary‐plane movements, suggesting these activities prioritize spinal stability over maximal spinal mobility, relying more on hip motion. This was expected since, in asymptomatic individuals, these functional tasks primarily engage the hips, while AR, LB, and FE predominantly involve the lumbar and thoracic spine. However, individuals with cLBP may find it more challenging to stabilize the spine during movement patterns due to pathology and deteriorated musculature in spinal regions and may slow down the overall motion. Despite coaching participants to keep their pelvis facing forward when performing bilateral movements, during CRF, for example, participants exhibited greater lumbar and hip excursion compared to thoracic excursion. These unexpected higher values could reflect compensatory movements, reduced proprioception, or other contributing factors affecting the movement patterns of individuals with cLBP.

Velocity and acceleration analyses for fast‐paced tests revealed distinct differences across spinal regions, tasks, and demographic groups, underscoring the complexity of spinal kinematics during both isolated and functional movements. Of the isolated movements, flexion exhibited the highest speeds and accelerations in the lumbar and hip regions. In contrast, the hip region showed comparatively lower velocities during AR and LB, reflecting its stabilizing function in these movements, while AR demonstrated the highest values for thoracic spine motion, which is consistent with the anatomical constraints and previous reports [17]. Among functional tasks, 5STS had the highest velocities and accelerations, particularly in the hip segment. Age‐related disparities were evident, with participants < 60 years old exhibiting more dynamic trunk and hip movements compared to participants ≥ 60 years old, particularly during functional tasks like 5STS.

A comparable study by Mageswaran et al. utilized similar methods to quantify spinal kinematics with Conity IMU sensors, measuring thoracolumbar ROM, velocity, and acceleration in participants with and without LBP [27]. Although the Conity sensors were not placed directly over specific spinal landmarks, the data can be generalized for comparisons to the trunk segment from this study. Their study, which included 50 LBP and 50 control participants (all under 65), reported axial plane ROM of 48.3°, lateral plane ROM of 45.0°, and sagittal plane (flexion) ROM of 34.7°. In comparison, the present study observed higher AR (64.7°) and LB (63.8°) values, while flexion ROM (36.5°) was similar in the trunk. Lower velocities and accelerations were reported within the present study, likely reflecting differences in methods, sample size, and demographic diversity. For instance, the LB^3^P cohort included a broader age range, while the Mageswaran study did not have any participants over age 65 and excluded those with a history of osteoporosis.

In a study conducted on the LB^3^P cohort with qualifying participants (*N* = 125), dynamic biplane radiography (DBR) was used to measure lumbar excursion during F/E and LB trials. While F/E comparisons are limited due to methodological differences, LB trials followed similar movement instructions, allowing for direct comparison. Kim et al. reported lumbar spine excursion values of 42.8° for F/E and 34.9° for LB [45]. This study's total F/E value of 45.6° aligns closely with the findings of Kim et al. despite participants in their study not necessarily performing maximal F/E. However, the total LB value of 18.3° in this study is lower, potentially due to IMU‐related skin artifact and/or differences in IMU placement compared to the DBR approach [40].

The magnitude of contributions for LPR revealed distinct motion strategies influenced by regional contributions. During the neutral‐to‐flexion range, participants tended to exhibit greater excursion within the hip region, with slightly larger ratios observed in the first quartile, followed by similar ratios across the remainder of the movement. This suggests that participants may have adopted a hip‐dominant movement pattern, with slightly greater lumbar contributions during the initial phase of flexion. In a recent review of the LPR literature, Vazirian et al. found that the magnitude of lumbar contribution is smaller in people with low back pain, which is consistent with the findings of the present study [46].

Male participants demonstrated lower LPR ratios than females across both neutral‐to‐flexion and neutral‐to‐extension, indicating greater hip dominance during these movements. While female participants also exhibited LPR ratios less than one during neutral‐to‐flexion (suggesting more hip contribution), their ratios were slightly higher than those of males, potentially reflecting a greater relative lumbar contribution to movement. Differences based on age groups were slightly more pronounced than those based on sex. Participants under 60 years had the largest LPR ratios for both movements, reflecting a relatively greater lumbar contribution compared to older participants. Vazirian et al. also found that the magnitude of lumbar contribution is smaller in the elderly and females, which is also consistent with the present study [46]. This could be attributed to age‐related changes in spinal or pelvic mobility, prompting older individuals to rely more on hip motion during trunk movements performed in the sagittal plane.

Relatively wide interquartile ranges observed across all quartiles (0%–25%, 25%–50%, 50%–75%, and 75%–100%) suggest heterogeneity in movement patterns among participants, likely reflecting differences in motor control strategies or musculoskeletal impairments within a large and diverse cohort. Variability in reported lumbopelvic rhythm ratios across studies is likely influenced by differences in LBP classifications and measurement methods. Deviations from these typical patterns may hold greater clinical significance, highlighting the interplay between lumbar and hip contributions during functional tasks and the clinical potential of these findings [11].

Due to resampling or streaming issues, data from certain sensors during specific tests were deemed unusable, which led to varying sample sizes for each test and segment. To minimize data limitations in future studies, more frequent checks for proper synchronization and calibration of sensors will be implemented during testing. Identification of unexpected movements relied on visual inspection of the movement signal by the authors, which is prone to human error. The integrated ROM method was successful in resolving unexpected movement patterns, likely resulting from inadequate calibration of the on‐board sensor fusion algorithm. Further development of this methodology may help to distinguish between sensor‐related artifacts and physiologically atypical movement patterns. Despite challenges such as sensor calibration, placement inaccuracies, and skin artifact, the BNO055 IMU sensors demonstrated their suitability for evaluating spinal and hip kinematics in the primary plane [47, 48, 49, 50, 51]. Future research should focus on three key areas: (1) further refinement of the procedures and algorithms to improve the wearability, usability, and reliability of the motion sensors, (2) translating laboratory measurements to clinical or home settings, and (3) identifying kinematic variables to phenotype and subgroup cLBP patients for targeted treatment.

A systematic review of lumbopelvic kinematics in participants with and without LBP concluded that “normative data may have limited relevance to a clinical environment unless the same measurement methods used to obtain published data are also used in the clinical setting where they are applied.” [17] Measuring spinal and hip kinematics reliably in clinical settings is often impractical due to cost, time, and resource constraints. The IMU‐based system utilized in this study is well suited to address these concerns due to the portable nature of the devices and associated mobile application, which provide step‐by‐step guidance and embedded data processing and visualization capabilities.

In conclusion, this study highlights the regional and demographic variability in spinal and hip motion dynamics. While the trunk dominates in isolated movements, the hip plays a critical role in functional tasks. Observed sex‐ and age‐related differences provide valuable insights into spinal and hip kinematics, with implications for future tailored rehabilitation strategies and performance optimization. The substantial variability observed within the same movements across all participants emphasizes the need for further phenotyping of individuals with cLBP to identify compensatory mechanisms or refine treatment plans. Future analyses will further leverage the richness of the LB3P dataset by incorporating inter‐repetition variability, motion phase segmentation (e.g., ascending vs. descending, LPR boundaries, etc.), and thoracic‐to‐lumbar contributions to provide a more granular understanding of regional coordination patterns. Moreover, while the current work focuses on reporting baseline kinematic patterns, future analyses will integrate comprehensive kinematic and patient‐reported outcome data across all study domains (including demographic factors) to identify distinct cLBP phenotypes, which may serve as a basis for predicting treatment response and guiding personalized interventions. Additionally, the variability in reported literature values underscores the importance of this study's large, diverse sample as a robust benchmark for future research and clinical applications.

## Conflicts of Interest

The authors declare no conflicts of interest.

## Supporting information


**Data S1:** STROBE Statement—checklist of items that should be included in reports of observational studies.
